# Prospective associations between diabetes and depressive symptoms across European regions: a secondary analysis of ELSA, TILDA, and SHARE datasets

**DOI:** 10.1093/eurpub/ckaf132

**Published:** 2025-09-29

**Authors:** Jaroslav Gottfried, Katarzyna Gajewska, Belinda Hernández, Rose Anne Kenny, Cathy Lloyd, Arie Nouwen, Shane O'Donnell, Ricardo Rodrigues, Norbert Schmitz, Sonya Deschênes

**Affiliations:** School of Psychology, University College Dublin, Dublin, Ireland; Diabetes Ireland, Dublin, Ireland; National Centre for Pharmacoeconomics, Dublin, Ireland; Department of Pharmacology and Therapeutics, Trinity Centre for Health Sciences, Trinity College Dublin, Dublin, Ireland; The Irish Longitudinal Study on Ageing, Trinity College Dublin, Dublin, Ireland; Discipline of Medical Gerontology, School of Medicine, Trinity College Dublin, Dublin, Ireland; Faculty of Wellbeing, Education & Language Studies, The Open University, Milton Keynes, United Kingdom; Department of Psychology, Middlesex University, London, United Kingdom; School of Business, University College Dublin, Dublin, Ireland; SOCIUS/CSG, ISEG (Lisbon School of Economics & Management), University of Lisbon, Lisbon, Portugal; Population-Based Medicine, University of Tübingen, Tübingen, Germany; School of Psychology, University College Dublin, Dublin, Ireland

## Abstract

This article investigates predictive associations between diabetes and depressive symptoms across Ireland, the United Kingdom, and four European regions. The data were obtained by merging datasets from three large prospective cohort studies—the English Longitudinal Study on Ageing, The Irish Longitudinal study on Ageing, and the Survey on Health, Ageing and Retirement in Europe. We first applied a survival analysis design to two samples of 43 061 and 35 993 participants, investigating elevated depressive symptoms as a risk factor for diabetes, and diabetes as a risk factor for elevated depressive symptoms, respectively. We next applied a multilevel modeling approach to examine depressive symptoms before, during, and after diabetes onset across 101 799 participants. We found a bidirectional association between diabetes and depressive symptoms; however, the strength of these associations did not significantly differ between the regions (*P* > .01). The results also showed that individuals with newly diagnosed diabetes consistently reported higher depressive symptoms than those without diabetes, even before diagnosis. However, we observed no country-specific differences in the gradual changes in depressive symptoms regardless of participants’ diabetes status. Diabetes at baseline was associated with higher risk of developing depression; and *vice versa*. These associations were not moderated by geographical location. Therefore, the risks of diabetes and depressive symptoms comorbidity seem to be equal across all observed geographic regions.

## Introduction

Major depression and elevated depressive symptoms are both associated with metabolic dysregulation, immune dysfunction, and cardiovascular complications [1]. Therefore, comorbidity between diabetes and depression is common. Longitudinally, depression increases the risk of diabetes, and diabetes increases the risk of depression [2–5]. However, the exact nature underlying this comorbidity requires further exploration.

One way to better understand diabetes and depression comorbidity is to study the heterogeneity in this association at the macrolevel. For instance, one study found differences in the prevalence of comorbid depression in people with type 2 diabetes were observed across continents, with Asia showing the highest prevalence and Europe the lowest [6]. Also, people with diabetes were found to be more likely to have depression if they were living in low- or middle-income regions, compared to high-income regions [7]. This notion was supported by Graham *et al*. [8] who, using a large European Social Survey sample of participants from 19 European regions, found that the likelihood of diabetes and depressive symptom comorbidity was higher when quality of diabetes care, measured by the Euro Diabetes Index [9], was low, suggesting that the quality of a country’s health infrastructure and system may play a meaningful moderating role.

## Study aims and hypotheses

Previous research findings suggest that the comorbidity of depressive symptoms in people with diabetes could be explained by societal, economic, or cultural factors [6–8]. Identifying these factors can inform country-specific prevention and healthcare policies, which would enhance the prevention and correct diagnosis of diabetes and depression comorbidity. We hypothesized that the strength of the prospective prediction of diabetes diagnosis based on depressive symptoms would be different across European regions (H1). Furthermore, we also hypothesized that the strength of the prospective prediction of depressive symptoms based on diabetes diagnosis would be different across European regions (H2). We also expected to observe country-specific differences in depressive symptoms before, during, and after the onset of diabetes (H3).

## Methods

### Sample

This study comprised the participants from three prospective cohort studies on ageing—the English Longitudinal Study on Ageing (ELSA) [10], The Irish Longitudinal Study on Ageing (TILDA) [11, 12], and the Survey on Health, Ageing and Retirement in Europe (SHARE) [13, 14]. We used data from five consecutive waves of collection from each cohort. Specifically, ELSA Waves 6–10 [15], which collected data from 2012 to 2023; TILDA Waves 1–5 [16–20], which collected data from 2009 to 2019; and SHARE Waves 4–8 [21–26], which collected data from 2011 to 2020. In each cohort, the data were collected in waves approximately every 2 years.

### Measures

#### Diabetes

Diabetes diagnosis was based on participant reports during a computer-assisted personal interview (CAPI). In the ELSA cohort, this was assessed via the question “*Has a doctor ever told you that you have diabetes?.*” In the TILDA cohort, this was assessed via the question “*Has a doctor ever told you that you have any of the conditions on this card?*” [*Diabetes or high blood sugar*]. In the SHARE cohort, participants were asked the question “*Has a doctor ever told you that you had/Do you currently have any of the conditions on this card?* [*Diabetes or high blood sugar*].”

#### Depressive symptoms

To measure depressive symptoms, TILDA uses the Center for Epidemiologic Studies Depression Scale (CES-D) [27] which originally included 20 Likert-type scale items in Waves 1–2, but was abbreviated to eight items in later cohorts. ELSA uses a different 8-item version of CES-D with binarized response options (yes/no), and SHARE uses the EURO-D European depression scale. The EURO-D scale was developed and validated for the purpose of international comparison, drawing inspiration from the CES-D, which results in a substantial content overlap in both measures [28]. Because of cohort measures using different depressive symptom scales, we applied *z*-score transformations to facilitate comparability across the cohorts.

#### Country/region

The cohorts include participants from Ireland (TILDA), the United Kingdom (ELSA), and other European regions (SHARE) grouped into the regions of *Northern Europe* (Denmark, Estonia, Finland, Latvia, Lithuania, Sweden), *Western Europe* (Austria, Belgium, France, Germany, Luxembourg, Netherlands, Switzerland), *Central and Eastern Europe* (Bulgaria, Croatia, Czech Republic, Hungary, Poland, Romania, Slovakia, Slovenia), and *Southern Europe* (Cyprus, Greece, Italy, Malta, Portugal, Spain), as per EuroVoc definition [29].

### Covariates

#### Body mass index

Body mass index (BMI) is expressed as a person’s weight in kilograms divided by their height in meters squared (kg/m^2^). According to the World Health Organization, normal BMI ranges from 18.5 to 25, with a BMI ≥ 25 considered as overweight, and ≥ 30 considered as obese [30].

#### Smoking

Smoking status was based on the participants’ self-report of whether they were currently active smokers (1) or not (0).

#### Alcohol drinking

ELSA measured the frequency of alcohol drinking in the last 12 months on a Likert-type scale with options ranging from *Almost every day* (1), through *Once or twice a month* (5), to *Not at all* (8). TILDA examined the frequency of alcohol drinking in the last six months on a Likert-type scale with options ranging from *Daily* (1), through *Once a week* (4), to *One or couple of days per year* (7). SHARE assessed the frequency of alcohol drinking by the number of days per week in the last three months when alcohol was consumed. The raw scores were reverse-coded and *z*-standardized, so higher scores indicated more frequent alcohol drinking.

#### Physical activity

In ELSA and SHARE, this was measured by the question “*Do you take part in sports or activities that are vigorous…*,” with the response options *More than once a week* (1), *Once a week* (2), *One to three times a month* (3), and *Hardly ever, or never* (4). Vigorous physical activity in TILDA was assessed as self-reported number of days per week. The raw scores were reverse-coded and z-standardized, so higher scores indicated more frequent physical activity.

### Analysis

Two discrete time survival analysis (Cox regression) models were used to test the Hypotheses 1 and 2. The first survival analysis estimated the time until the onset of diabetes (or censoring) based on the severity of depressive symptoms and country/region of living. The second survival analysis estimated the time until the occurrence of a higher severity of depressive symptoms, cut-off at +1 *z*-score, based on diabetes status and country/region of living. Baseline models with only age and gender as the predictors were first examined. Then, we added depressive symptoms/diabetes (depending on the direction of the model) and country/region of living in the models. Next, we included the interaction between baseline diabetes/depression status and country/region. Finally, we included the additional covariate variables of BMI, smoking, alcohol consumption, and physical activity. Baseline consisted of Wave 6 for ELSA, Wave 1 for TILDA, and Wave 4 for SHARE.

Hypothesis 3 was tested by mixed linear modeling. We aimed to predict the level of depressive symptoms (continuous depressive symptom *z*-scores) based on a new diabetes diagnosis, the country/region of living, and the time period relative to the onset of diabetes, classified into three categories—*before* (one data collection wave before diabetes onset, coded as −1), *recent* (the data collection wave in which diabetes was recorded for the first time, coded as 0 and serving as the reference category), and *after* (one data collection wave after diabetes onset, coded as 1). For the sample without diabetes, we designed the *before* time period as the first wave in which data on diabetes were recorded for each participant, *recent* as the next data collection wave after that, and *after* as the next wave after that.

In the mixed linear model, the level of depressive symptoms at different times was predicted by diabetes, country/region of living, and their interactions, alongside the control variables of gender, age, BMI, smoking, and physical activity. Based on the survival model results (see below), alcohol consumption was not included as a covariate, because of its likely nonlinear relationship with depression. In the baseline model, we included gender and age as fixed effect predictors and participant ID as the random effect. Next, we added the country/region of living and the diabetes status groups as fixed effect predictors to the model, and the mutual interactions between the time period, diabetes, and the country/region of living in the next step. Finally, for the final model, we dropped the statistically non-significant interaction terms and added the covariate variables of BMI, smoking, and physical activity.

In all analyses, we designed the region of Western Europe to be the reference country/region category because of its largest sample size. Model fit was compared using the log-likelihood ratio tests (LRT). Due to the substantial sample size, we set the level of statistical significance for the predictors and the LRT to a slightly stricter threshold of *P* ≤ .01. The analyses were conducted in R [31].

## Results

### Hypothesis 1

#### The strength of the prospective prediction of diabetes diagnosis based on depressive symptoms is different across European regions

The final sample included 43 061 participants (57% female) with a mean age of 65.2 years (SD = 9.4). Sample characteristics are presented in Supplementary Table S1.

#### Time to onset of diabetes

Over the course of the follow-up period, 2532 (6%) participants developed diabetes. The overall diabetes incidence rate was approximately one per 100 person-years. In the baseline model with age and sex as the only predictors, female gender was found to be a protective factor against the onset of diabetes (HR = 0.75, *P* < .001), and higher age was found to be a weak, but still significant risk factor (HR = 1.02, *P* < .001). Adding the country/region of living and the severity of depressive symptoms significantly improved the model, as showed by LRT test results, *χ*^2^(6) = 385.3, *P* < .001. With Western Europe as the reference region, participants from Ireland were at a lower risk of diabetes (HR = 0.72, *P* < .001). We found a heightened risk of diabetes among people living in the UK (HR = 1.24, *P* = .009), Northern Europe (HR = 1.27, *P* < .001), Central/Eastern Europe (HR = 1.99, *P* < .001), and Southern Europe (HR = 2.13, *P* < .001), compared to Western Europe.

**Figure 1.**
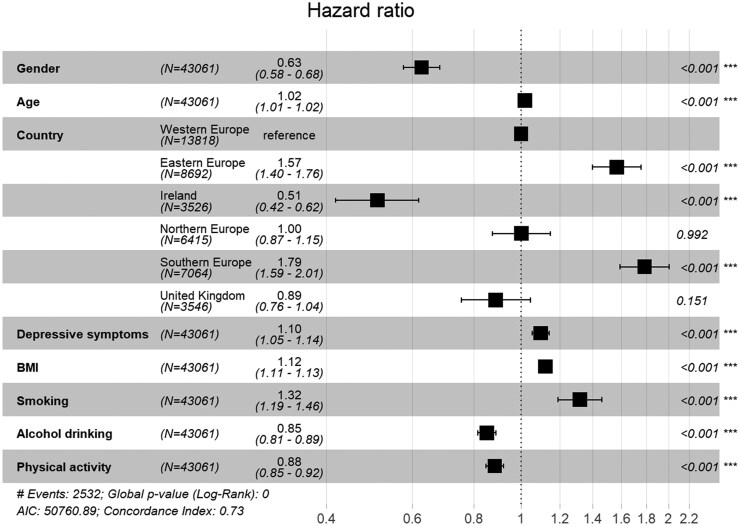
Forest plot demonstrating the effect estimates and their 95% CIs for predicting the onset of type 2 diabetes.

In the second model, more severe depression symptoms were associated with an earlier onset of diabetes (HR = 1.18, *P* < .001). That is, participants’ risk of developing diabetes within each time interval rose by nearly one fifth for each *z*-score in depression symptoms at baseline. In the next step, we added the interaction effect between country/region and depressive symptoms to the model. This model was not found to be substantially better (*P* = .139), and none of the interaction terms reached the statistical significance threshold of *P* < .01. Ultimately, we discarded the interaction terms and added the control variables of BMI, smoking, alcohol drinking, and physical activity in the final model, which resulted in a major fit improvement over the first model, *χ*^2^(4) = 1058.4, *P* < .001. Figure 1 summarizes the results of the final model. As shown in Fig. 1, higher BMI and smoking are considerable risk factors for diabetes onset, whereas alcohol and participating in physical activities seem to be protective factors. Even with the control variables, depressive symptoms retained a modest amount of predictive power (HR = 1.10, *P* < .001). The risk of diabetes generally increased with more severe depressive symptoms (detail in Supplementary Fig. S2), however there was no strong support for different hazard ratios across the geographical regions. Thus, our results do not support Hypothesis 1, indicating that diabetes–depression associations remain stable across regions.

### Hypothesis 2

#### The strength of the prospective prediction of elevated depressive symptoms based on diabetes diagnosis is different across European regions

The final sample for the analysis comprised 35 993 participants (53% female) who were 65.4 (SD = 9.2) years of age at baseline, on average. Sample characteristics are presented in Supplementary Table S2.

#### Time to elevated depressive symptoms

The overall incidence of elevated depressive symptoms in the sample was approximately 6.63 per 100 person-years. In the baseline model, female gender was associated with a higher risk of elevated depressive symptoms (HR = 1.42, *P* < .001). Older age was also a statistically significant predictor, but with a small effect size (HR = 1.01, *P* < .001). Adding country/region of living and the presence of diabetes at baseline as predictors significantly improved the model [*χ*^2^(6) = 1215.8, *P* < .001]. With Western Europe as the reference region, participants from the UK and Ireland were at an overall lower risk of developing elevated depressive symptoms (HR = 0.54, *P* < .001, and HR = 0.66, *P* < .001, respectively). Conversely, earlier onset of elevated depressive symptoms occurred in participants from Northern Europe (HR = 1.25, *P* < .001), Central/Eastern Europe (HR = 1.30, *P* < .001), and, particularly, Southern Europe (HR = 1.65, *P* < .001). Having diabetes at baseline was associated with an increased risk of elevated depressive symptoms (HR = 1.29, *P* < .001).

**Figure 2.**
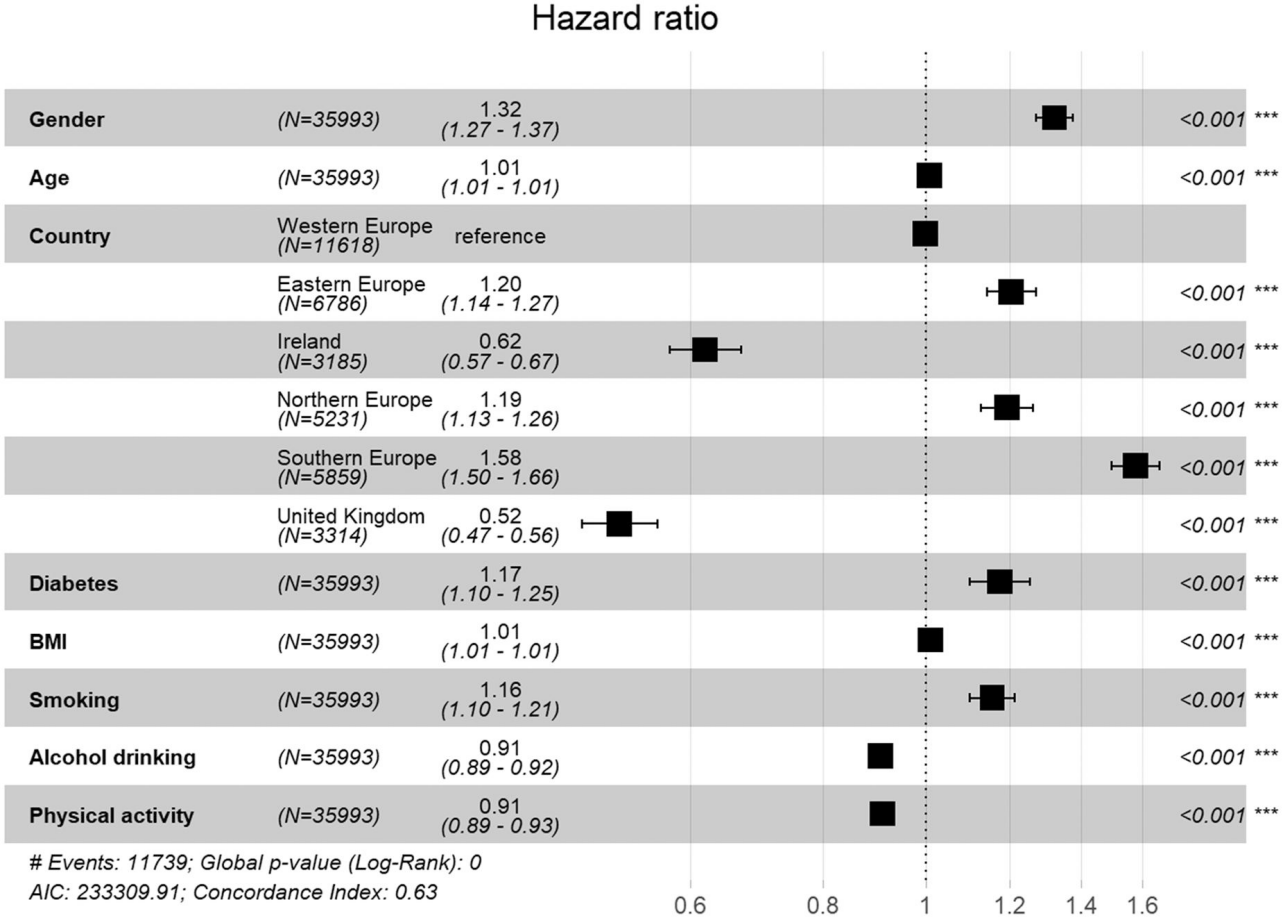
Forest plot demonstrating the effect estimates and their 95% CIs for predicting the onset of elevated depressive symptoms.

In the next step, we added the interaction effect between the country/region of living and diabetes status to the model. This second model was not substantially better than the first one (*P* = .346), and none of the interaction terms reached the statistical significance level of *P* < .01. Ultimately, we discarded the country/region and diabetes interaction and added the control variables of BMI, smoking, alcohol consumption, and physical activity in the final model, which resulted in an improvement in prediction over the first model [*χ*^2^(4) = 236.2, *P* < .001]. Figure 2 demonstrates that higher BMI and, especially, smoking are risk factors for elevated depressive symptoms. On the other hand, drinking alcohol and participating in physical activities seem to be protective factors. Even with the potential effects of control variables considered, diabetes remained a modest unique risk factor for the onset of elevated depressive symptoms—it raised the individual risk of scoring high on depressive symptoms by about one fifth (HR = 1.17, *P* < .001). Supplementary Fig. S2 demonstrates graphically that people with diabetes experienced elevated depressive symptoms more often, though this did not vary significantly across country/region groups. Consequently, the results do not support Hypothesis 2.

### Hypothesis 3

#### There are country/region-specific differences in depressive symptoms before, during, and after the onset of diabetes

The final sample for this analysis comprised 101 799 participants. In the initial statistical model, depressive symptoms were predicted by participant’s gender, age at the first time of data collection, and time period. Higher age predicted slightly higher level of depressive symptoms (*B* = .01, *P* < .001), and women tended to report considerably more depressive symptoms compared to men (*B* = .33, *P* < .001). Participants who did not develop diabetes overall reported the highest levels of depressive symptoms the first time they were assessed (*B* = .05, *P* < .001). Across the entire sample, there were no substantial differences in depressive symptoms between the second and the third time period (*P* = .502). The best improvement of the model occurred in the first step when country/region and diabetes were added as separate predictors. Although all models showed statistically significant improvement, the Bayesian information criterion (BIC) indicators suggested the improvement of further models was small (details in Supplementary Table S3). This was supported by the fact that none of the newly added interaction predictors were statistically significant (*P* > .01) in models 3 and 4. Consequently, we excluded the non-significant interactions before proceeding to adding the control variables (BMI, smoking, and physical activity) and build the final model.

**Figure 3.**
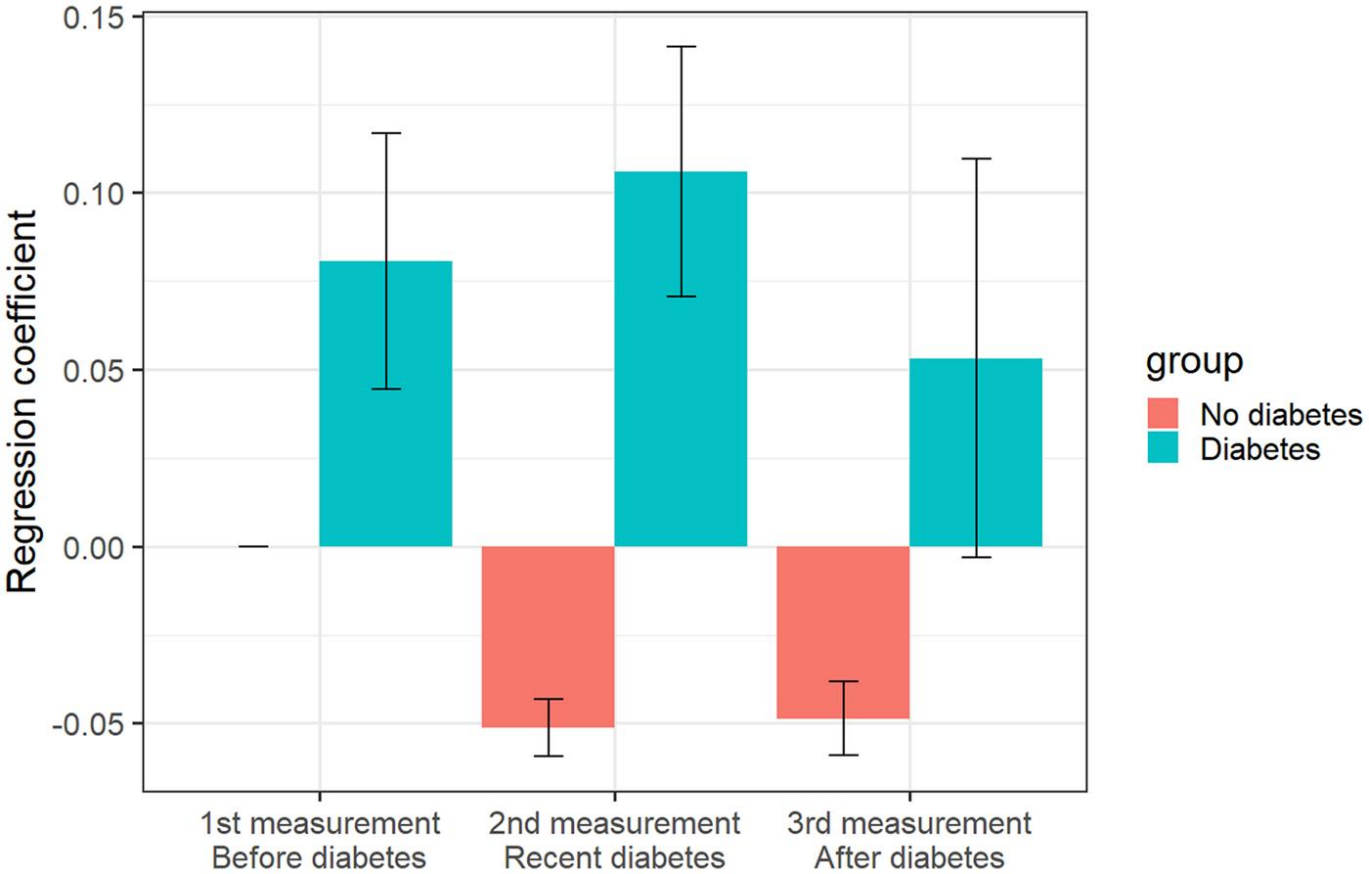
Linear mixed effect model coefficients of time periods on the depressive symptoms z-score grouped by diabetes presence with 95% CIs.

Table 1 contains results of the final model and Fig. 3 presents a graphical comparison of the regression estimates for diabetes non-diabetes groups across time with 95% CIs. Specifically, compared to the Western Europe region, participants from Northern Europe reported more severe depressive symptoms (*B* = .10, *P* < .001), as well as participants from Southern Europe (*B* = .05, *P* < .001) and Ireland (*B* = .04, *P* < .001). On the other hand, there were no substantial differences between participants from Western Europe and those from Eastern Europe (*P* = .147) or the United Kingdom (*P* = .156). People who developed diabetes reported higher levels of depressive symptoms (*B* = .08, *P* < .001) overall, especially at the time of diabetes onset (*B* = .16, *P* < .001).

**Table 1.** Final linear mixed model results

Predictor	*B*	SE	*t*	*P*
(Intercept)	−0.99	0.027	−37.4	<.001
Gender (female)	0.33	0.006	59.1	<.001
Age	0.01	0.001	30.3	<.001
Measurement (second)	−0.05	0.004	12.4	<.001
Measurement (third)	−0.05	0.005	−9.1	<.001
Northern Europe	0.10	0.009	11.6	<.001
Eastern Europe	0.01	0.008	1.5	.147
Southern Europe	0.05	0.007	7.1	<.001
Ireland	0.04	0.011	4.0	<.001
United Kingdom	0.02	0.013	1.4	.156
Diabetes	0.08	0.018	4.4	<.001
BMI	0.01	0.001	10.7	<.001
Smoking	0.15	0.007	21.1	<.001
Physical activity	−0.11	0.002	−49.3	<.001
Measurement (second) * Diabetes	0.08	0.021	3.7	<.001
Measurement (third) * Diabetes	0.02	0.030	0.7	.475

Because no interaction term including country/region of living was statistically significant, we conclude we did not find support for Hypothesis 3. However, the results demonstrated that people with diabetes tend to report more severe depressive symptoms than people without diabetes before, during, and after the diagnosis.

## Discussion

The results showed that having elevated depressive symptoms at baseline was a risk factor for diabetes incidence, and having diabetes at baseline was a risk factor for elevated depressive symptoms. Specifically, having diabetes was associated with a 17% higher risk of elevated depressive symptoms in the future; and a rise in depressive symptoms by one standard deviation was associated with a 10% higher risk of developing diabetes in the future, after controlling for several sociodemographic and lifestyle/health covariates. These findings are in line with existing literature on the associations between diabetes and depression [2, 4, 5, 32–35] and serve as further support for the existence of a robust association between diabetes and depressive symptoms.

However, we found no strong evidence supporting hypotheses 1 and 2 which proposed regional differences in associations between diabetes and depressive symptoms. This suggests that the association between diabetes and depressive symptoms might be stable regardless the institutional setting and different underlying risk rates across different European regions, Ireland, and the United Kingdom. The country/region level of depressive symptoms or diabetes does not seem to change the strength of their association, which is a plausible conclusion, because a change in prevalence does not necessarily cause a change in association. Although we did find notable differences between regions in the risk of developing both diabetes and elevated depressive symptoms, it was difficult to interpret the regional differences, because there was considerable variation in depressive symptoms between regions even within each regional group. Such country/region-level variation was also recently found in the large-scale comparison of European regions by Arias-de la Torre *et al*. [36].

Interestingly, participants in Ireland were found to be at lower risk of diabetes and elevated depressive symptoms compared to Western Europe in the survival analyses but were at higher risk of elevated depressive symptoms in the mixed linear model. This incongruency was explained *post hoc* by finding out that the overall level of depressive symptoms in the Ireland sample raised substantially in the last three waves collected in 2014–2015, 2016, and 2018, respectively. The mixed linear model analysis sample consisted of substantially more people from later years which on average exhibited more depressive symptoms in comparison to participants from Western Europe whose depressive symptom scores did not increase in the last three waves of data collection.

Because we did not find evidence that the strength of the association between diabetes and depressive symptoms differs across European regions, even though diabetes and depressive symptoms prevalence did, we hypothesize there are other than geographical factors which moderate this relationship. The most likely predictors might be related to socioeconomic, healthcare, and cultural policies, including the quality of diabetes-related healthcare in a country, which might predict the extent to which diabetes affects mental health. Alternatively, it is possible that the diabetes and depressive symptoms association is stable regardless of these factors. Future research should explore whether the factors of healthcare quality, socioeconomic status, and mental health awareness contribute to variations either in the prevalence or the strength of the diabetes and depressive symptoms association.

### Limitations

In SHARE and TILDA data, it was not possible to easily distinguish between the diagnosis of diabetes and hyperglycemia, as the participants were asked about both medical conditions within one question. Similarly, we could not distinguish between diabetes types.

There are also some comparability issues with CES-D and EURO-D scores. Both scales were found to be strongly correlated, but their agreement moderate, with some systematic discrepancies [37]. Because we found non-significant results even between regions measured exclusively with EURO-D, we find it unlikely that comparability issues challenge our study findings.

### Conclusions and implications

We found that the strength of the association between diabetes and depressive symptoms did not significantly vary across Ireland, the United Kingdom, and European regions, despite differences in healthcare quality and socioeconomic factors. Common public health policies across regions have often been limited in face of different associations between symptoms across regions. The findings underscore that diabetes and depressive symptoms may be an exception to this and show potential for Europe-wide shared public health policies to integrate mental health screenings into routine diabetes care. They seem to indicate that public health efforts to tackle diabetes and depression comorbidity may be applicable across Europe and do not necessarily need to be tailored for each country specifically beyond the adjustment for the local rate of diabetes and depression incidence. Future public health strategies across Europe should consider the challenge of depression and diabetes comorbidity.

## Supplementary Material

ckaf132_Supplementary_Data

## Data Availability

The data underlying this article were provided by SHARE, ELSA, and TILDA projects. The analytic script with detailed result output is available at https://osf.io/gx9kq/ Key points Diabetes and depressive symptoms are correlated, and risk of their comorbidity is elevated. Comorbidity between diabetes and depressive symptoms seems to behave the same way across most European countries. While people with diabetes are generally more likely to develop depression and *vice versa*, the comorbidity risks seem to be stable regardless of the European region, after accounting for diabetes and depression prevalence rates and individual-level factors.
