# Supplementary material for: Prospective associations between diabetes and depressive symptoms across European regions: a secondary analysis of ELSA, TILDA, and SHARE datasets
Source: Eur J Public Health. 2025 Sep 29;35(6):1264–70. doi: 10.1093/eurpub/ckaf132 (PMC12707472; doi:10.1093/eurpub/ckaf132)

**Supplementary Materials**

**Table S1**

*First analysis sample characteristics*

|  |  | | *n* | % | *M*(*SD*) |
| --- | --- | --- | --- | --- | --- |
| Age | |  | 43 061 |  | 65.2(9.4) |
| Gender | Male | | 18 716 | 43.5 |  |
|  | Female | | 24 345 | 56.5 |  |
| Education | Primary | | 15 222 | 35.6 |  |
|  | Secondary | | 17 695 | 41.4 |  |
|  | Tertiary | | 9838 | 23.0 |  |
| Marital status | Married/living together | | 30 226 | 70.2 |  |
|  | Divorced/separated | | 4600 | 10.7 |  |
|  | Single/no marriage | | 2422 | 5.6 |  |
|  | Widowed | | 5800 | 13.5 |  |
| Country/region | Western Europe | | 13 818 | 32.1 |  |
|  | Eastern/Central Europe | | 8692 | 20.2 |  |
|  | Northern Europe | | 6415 | 14.9 |  |
|  | Southern Europe | | 7064 | 16.4 |  |
|  | Ireland | | 3526 | 8.2 |  |
|  | United Kingdom | | 3546 | 8.2 |  |
| BMI | | | 43 061 |  | 26.8(4.4) |
| Smoking | | | 7955 | 18.5 |  |
| Self-perceived health*** | | | 43 053 |  | 3.0(1.1) |

***Measured on a 5-point scale, ranging from 1 - poor to 5 - excellent.

**Table S2**

*Second analysis sample characteristics*

|  |  | | *n* | % | *M*(*SD*) |
| --- | --- | --- | --- | --- | --- |
| Age | |  | 35 993 |  | 65.3(9.2) |
| Gender | Male | | 16 931 | 47.1 |  |
|  | Female | | 19 062 | 53.0 |  |
| Education | Primary | | 12 204 | 34.1 |  |
|  | Secondary | | 15 033 | 42.1 |  |
|  | Tertiary | | 8505 | 23.8 |  |
| Marital status | Married/living together | | 25 700 | 71.4 |  |
|  | Divorced/separated | | 3711 | 10.3 |  |
|  | Single/no marriage | | 2008 | 5.6 |  |
|  | Widowed | | 4562 | 12.7 |  |
| Country/region | Western Europe | | 11 618 | 32.3 |  |
|  | Eastern/Central Europe | | 6786 | 18.9 |  |
|  | Northern Europe | | 5231 | 14.5 |  |
|  | Southern Europe | | 5859 | 16.3 |  |
|  | Ireland | | 3185 | 8.8 |  |
|  | United Kingdom | | 3314 | 9.2 |  |
| BMI | | | 35 993 |  | 27.0(4.5) |
| Smoking | | | 6372 | 17.7 |  |
| Self-perceived health*** | | | 35 987 |  | 3.1(1.0) |

***Measured on a 5-point scale, ranging from 1 - poor to 5 - excellent.

**Table S3**

*Nested model comparison*

| Model no. | Added predictors | BIC | *χ*2 | df | *p* |
| --- | --- | --- | --- | --- | --- |
| Baseline | Gender + Age + Time | 574 814 |  |  |  |
| 1 | Country/region + Diabetes | 574 551 | 337.16 | 6 | < .001 |
| 2 | Time * Diabetes | 574 553 | 23.157 | 2 | < .001 |
| 3 | Country/region * Diabetes | 574 591 | 23.072 | 5 | < .001 |
| 4 | Time * Diabetes * Country/region | 574 719 | 118.03 | 20 | < .001 |

**Figure S1**

*Predicted type 2 diabetes risk scores and their 95% confidence intervals based on country/region of living and level of depression symptoms*


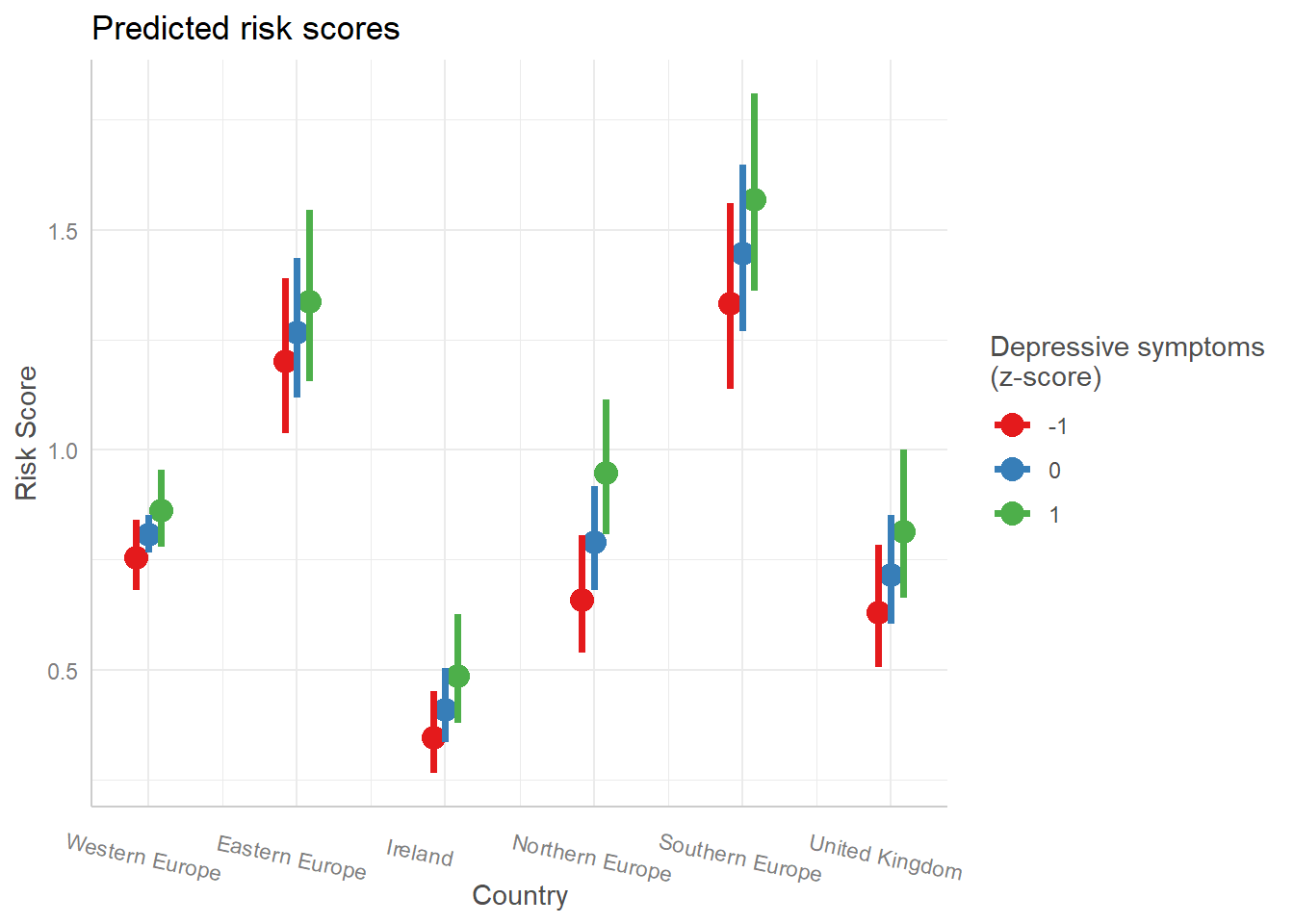


**Figure S2**

*Predicted elevated depressive symptom risk scores and their 95% confidence intervals based on country/region of living and diabetes status*


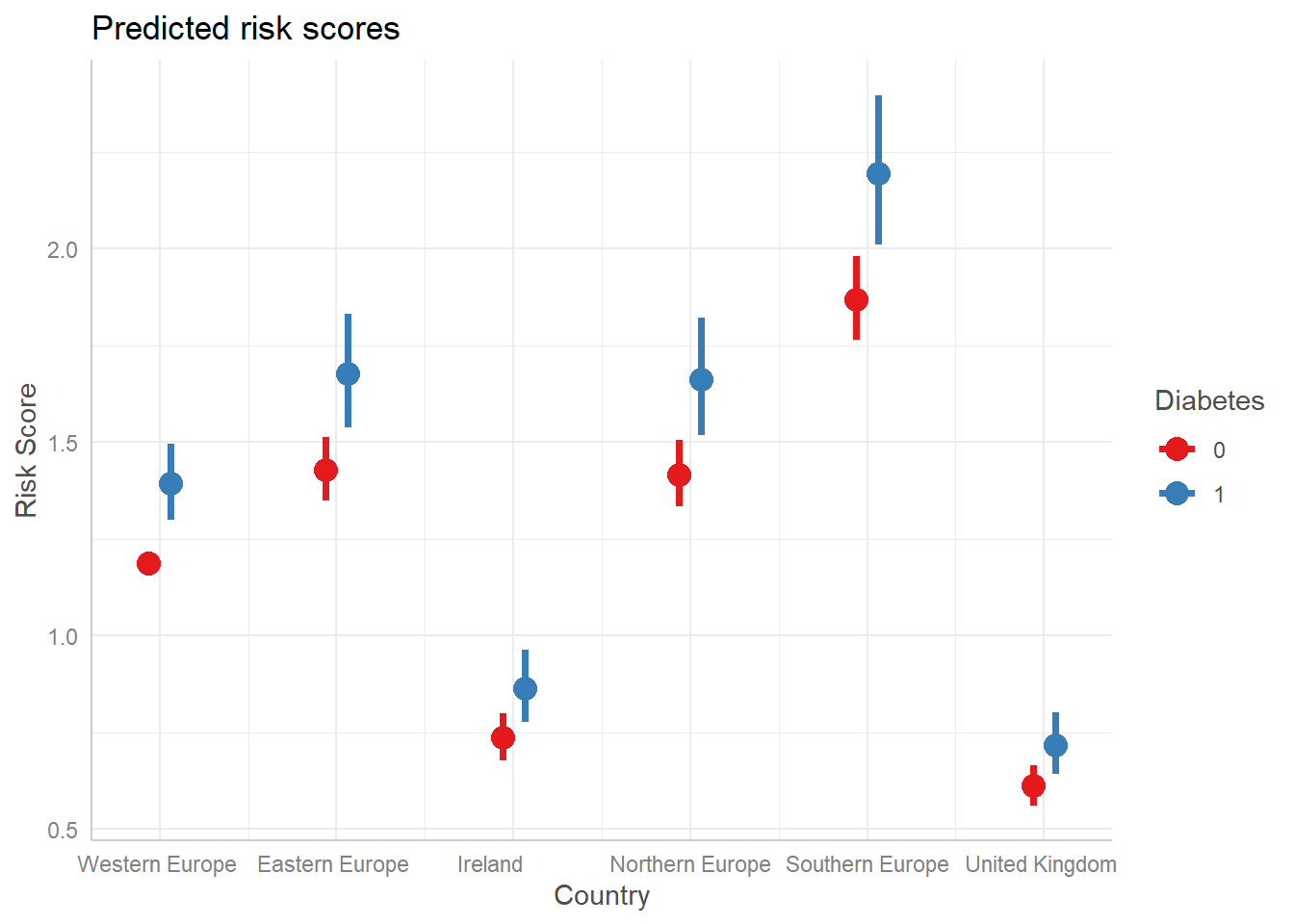

Supplement: ckaf132_Supplementary_Data [file ckaf132_supplementary_data.docx]
